# Supplementary material for: ED-B-Containing Isoform of Fibronectin in Tumor Microenvironment of Thymomas: A Target for a Theragnostic Approach
Source: Cancers (Basel). 2022 May 24;14(11):2592. doi: 10.3390/cancers14112592 (PMC9179240; doi:10.3390/cancers14112592)
Supplement: Supplementary file 1 [file cancers-14-02592-s001.zip › cancers-1693399-supplementary.pdf]

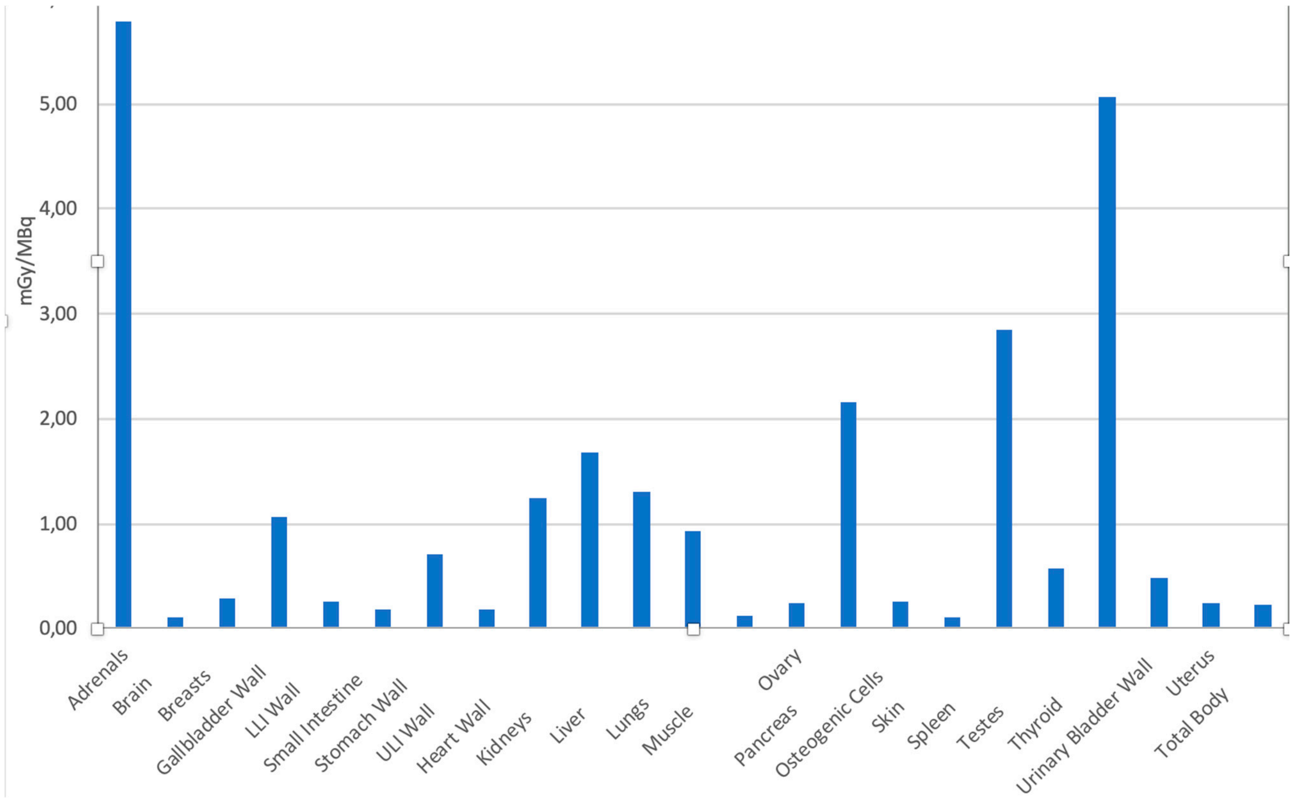

Supplementary Figure S1. Average estimated absorbed doses of beta radiation to healthy organs calculated by Olinda.

AMt

NF

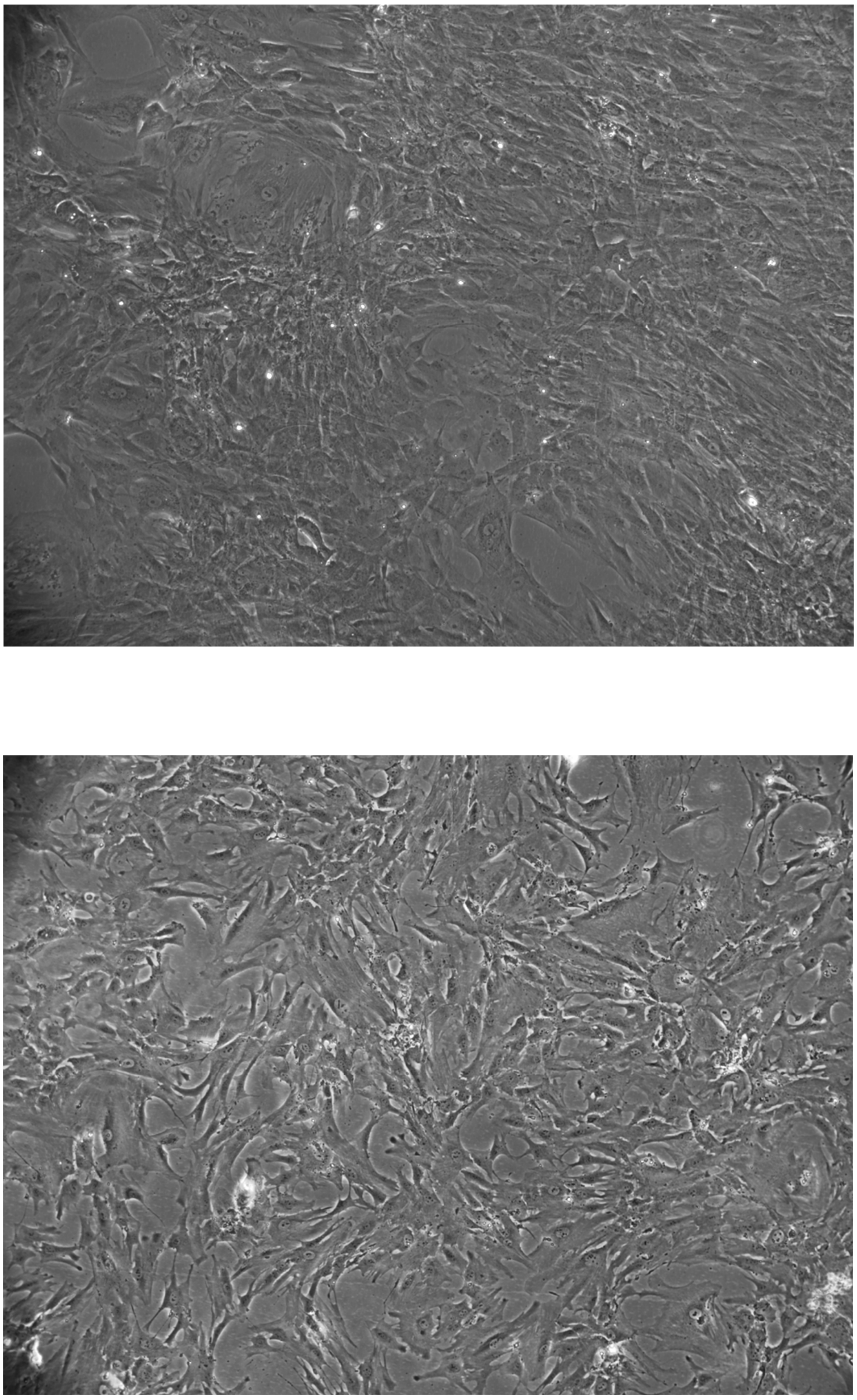

Figure S2: Morphology of AMt and NF primary cell culture at passage 1. Pictures were taken by phase contrast microscopy.

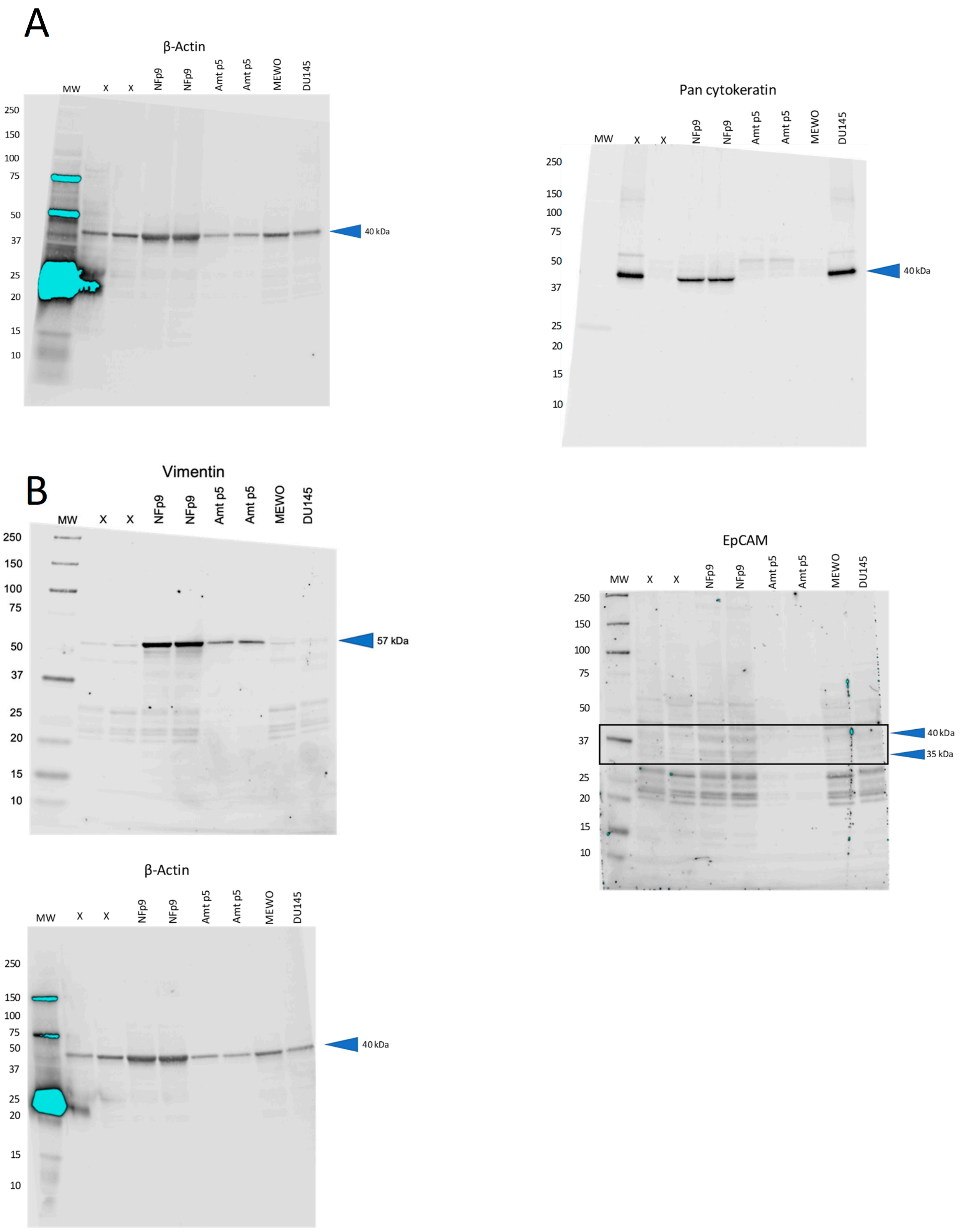

Supplementary Figure S3 – Western blotting evaluation of EpCAM, Vimentin and Pan cytokeratins expression in NF and AMt cells.

(A) Western blotting was performed using anti-Pan cytokeratin primary antibody (Abcam, Cambridge, UK) 1:300, anti-Vimentin (Cell Signaling Technology, Massachussets, USA) 1:1000 and Rhodamine anti-Actin (Biorad, Hercules, California, USA) 1:2500 as housekeeping protein. Results confirmed Vimentin expression in NF and AMt cells, whereas Pan cytokeratins expression was confirmed in NF cells only. (B) Western blotting did not reveal any expression of EpCAM in the evaluated cells (see box marked in black colour). Anti-EpCAM (Abcam, Cambridge, UK) 1:1000 primary antibody

and Rhodamine anti-Actin (Biorad, Hercules, California, USA) 1:2500 was used. DU145 and MEWO cell lines were used as controls.
X: samples not included in the evaluation

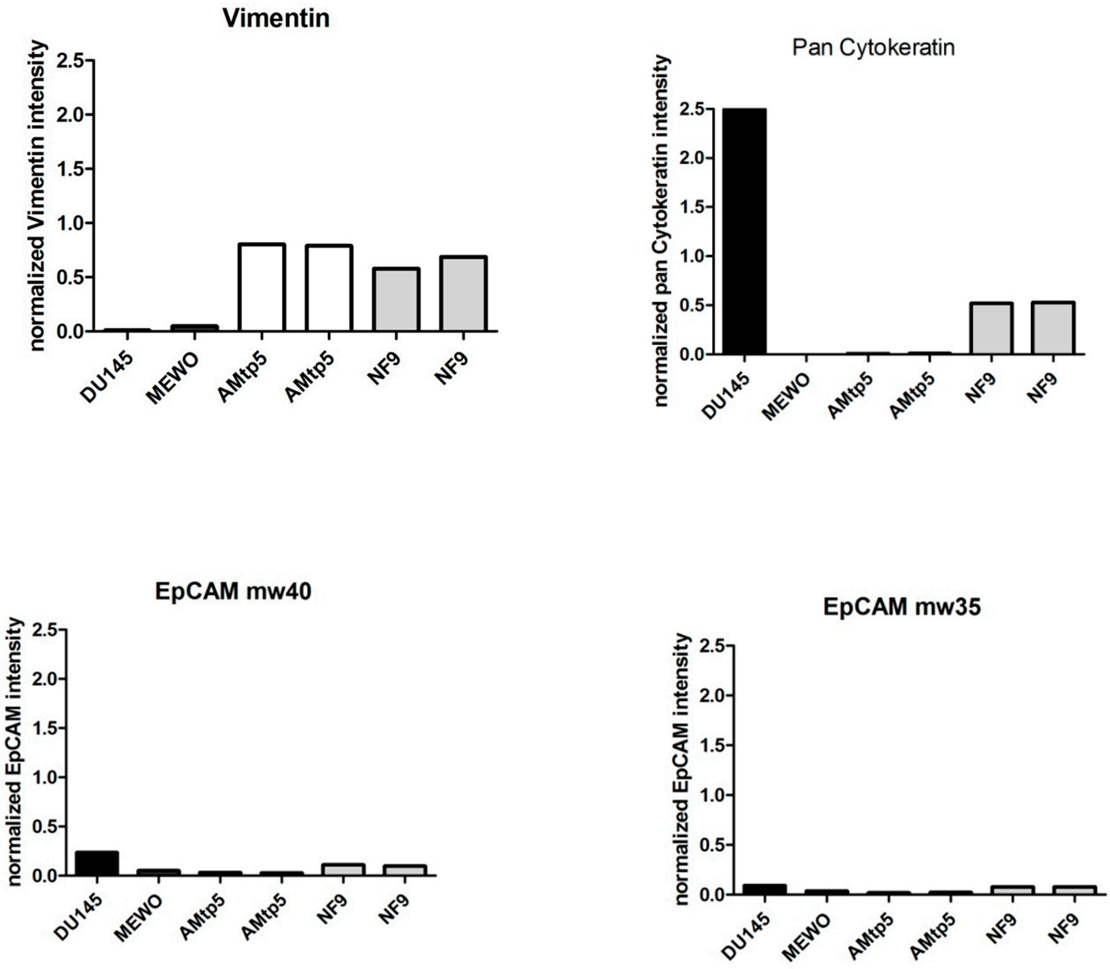

Supplementary Figure S4 – Densitometry analysis of EpCAM, Vimentin and Pan cytokeratins expression in NF and AMt cells. Adjusted intensity volumes were normalized referring to β-Actin values using ImageLab software (Biorad).

Supplemental Table S1. Patients' baseline characteristics

	All Patients n = 11
Median age, years - range	53 (32-63)
Gender	
Male	9
Female	2
Tumor stage at diagnosis, Masaoka*	
I	1
II	0
III	1
IV	8
Previous therapy	
Surgery	7
Intracavitary hypertermic chemoteraphy	3
Chemotherapy, n > 3	4
Radiotherapy	5
Somatostatin analougues	11
*In one patient both tumor stage and histological type were unknow	

Supplemental Table S2. calculated dosimetric estimates for target lesion(s) and bone marrow

Pt	Target lesion			Bone Marrow	
	Side	Adsorbed dose (mSv/MBq)	Provisional dose (Gy)*	Adsorbed dose (mSv/MBq)	Provisional dose (Gy)*
44014	Liver	1.78	5.99	0.172	0.57
44026	Pleura	8.1	44.95	0.103	0.57
44031	Liver	0.2	1.11	0.167	0.93
44032	Pleura	0.6	3.33	0.0939	0.52
G004	Liver	7.2	58.32	0.2	1.63
G007	Thymic lodge	9.8	102.3	0.308	2.6
G016	Thymic lodge	18.7	192.98	0.255	2.36
G025	Liver	1.8	16.98	0.369	3.48
	Liver	1.7	16.04		
G030	Paratracheal lymph node	0.3	2.34	0.331	2.58
SW	Xx	Xx	Xx	Xx	xx
CS	Pleura	Xx	Xx	Xx	xx

* Calculated for the effective administered activity for treated patients, and for the hypothetical administered activity for untreated patients based on cohort RIT.
